# *Journal of Functional Biomaterials* Best Paper Award 2015

**DOI:** 10.3390/jfb6020395

**Published:** 2015-06-09

**Authors:** Francesco Puoci

**Affiliations:** Dipartimento di Scienze Farmaceutiche, Università della Calabria, Edificio Polifunzionale, Arcavacata di Rende (CS) 87036, Italy; E-Mail: francesco.puoci@unical.it

The *Journal of Functional Biomaterials* is instituting an annual award to recognize the outstanding papers related to materials for biomedical applications published in this journal. We are pleased to announce the first “*Journal of Functional Biomaterials* Best Paper Award” for 2015.

Nominations were made by the Editorial Office of *Journal of Functional Biomaterials* based on the citations and downloads among all the research papers published in 2014 and 2013. We are pleased to announce that the following three papers were chosen by the Prize Awarding Committee:

**Best Paper Award**
  1st Prize
    **Victoria Leszczak, Dominique A. Baskett and Ketul C. Popat**
    Smooth Muscle Cell Functionality on Collagen Immobilized Polycaprolactone Nanowire Surfaces
    *J. Funct. Biomater.* **2014**, *5*(2), 58–77; doi:10.3390/jfb5020058
    Available online: http://www.mdpi.com/2079-4983/5/2/58
    “*A very comprehensive study of an important cell–material interaction that could be of wide interest to tissue engineers.*”––Prof. Dr. Joachim B. Kohn
      “*Advanced biomaterial concepts, comprehensive characterization.*”––Prof. Dr. Aldo R. Boccaccini
      “*A very accurate biological characterization.*”––Dr. Antonella Sola
      “*Useful study of SMC morphology and turnover on a surface of nanowires orientated vertically to the surface. Normally such structures are horizontally arrayed on the surface. More could have been done to rationalise the structure with a surface that has so many unusually deep vacant spaces. The physiological relevance of such an unusual surface is also not clear*.”––Prof. Dr. Pankaj Vadgama
  2nd Prize
    **Adel M.F. Alhalawani, Declan J. Curran, Belinda Pingguan-Murphy, Daniel Boyd and Mark R. Towler**
    A Novel Glass Polyalkenoate Cement for Fixation and Stabilisation of the Ribcage, Post Sternotomy Surgery: An *ex-Vivo* Study
    *J. Funct. Biomater.* **2013**, 4(4), 329–357; doi:10.3390/jfb4040329
    “*The article describes an interesting result but is only of limited interest to a specialized subgroup of scientists*.”––Prof. Dr. Joachim B. Kohn
      “*Comprehensive, scientifically sound, well written and completed by a rich bibliography*.”––Dr. Antonella Sola
      “*Exceptionally comprehensive study of based glass polyalkenoate cements with comparison of surface, bulk and ion release properties. It has breadth and sufficient bone fixation interest to be of significance to all who work in this field. However, it tends to veer towards the descriptive rather than a mechanistic analysis*.”––Prof. Dr. Pankaj Vadgama
  3rd Prize
    **Deepen Paul, Sharmistha Paul, Nima Roohpour, Mark Wilks and Pankaj Vadgama**
    Antimicrobial, Mechanical and Thermal Studies of Silver Particle-Loaded Polyurethane
    *J. Funct. Biomater.* **2013**, *4*(4), 358–375; doi:10.3390/jfb4040358
    Available online: http://www.mdpi.com/2079-4983/4/4/358
    “*The article describes an interesting result but is only of limited interest to a specialized subgroup of scientists. It’s a well done study.*”––Prof. Dr. Joachim B. Kohn
      “*Relevant, timely, well conducted study on Ag nanoparticles as part of a composite material.*” ––Prof. Dr. Aldo R. Boccaccini
      “*A step forward into the investigation of Ag use for antibacterial applications.*”––Dr. Antonella Sola

These three outstanding papers are valuable contributions to the *Journal of Functional Biomaterials*. On behalf of the Prize Awarding Committee and the Editorial Board, we would like to congratulate these three teams for their excellent work. In recognition of their accomplishment, the corresponding authors of these three best papers, Dr. Ketul C. Popat, Dr. Mark R. Towler, Dr. Deepen Paul and Dr. Pankaj Vadgama will receive the privilege of publishing an additional paper free of charge in open access format in the *Journal of Functional Biomaterials* after regular peer-review procedures.

**Prize Awarding Committee**

Editor-in-Chief

Prof. Dr. Francesco Puoci

Dipartimento di Scienze Farmaceutiche, Università della Calabria, Edificio Polifunzionale, Arcavacata di Rende (CS) 87036, Italy

E-Mail: francesco.puoci@unical.it

Associate Editor

Prof. Dr. Pankaj Vadgama

Interdisciplinary Research Centre (IRC) in Biomedical Materials, Queen Mary, University of London, Mile End Road, London E1 4NS, UK

E-Mail: p.vadgama@qmul.ac.uk

Editorial Board Member

Prof. Dr. Aldo R. Boccaccin

Institute of Biomaterials, Department of Materials Science and Engineering, University of Erlangen-Nuremberg, 91058 Erlangen, Germany

E-Mail: aldo.boccaccini@ww.uni-erlangen.de

Editorial Board Member

Prof. Dr. Joachim B. Kohn

The New Jersey Center for Biomaterials, Rutgers, The State University of New Jersey, 145 Bevier Rd., Piscataway, NJ 08854, USA

E-Mail: kohn@rutgers.edu

Editorial Board Member

Prof. Dr. Dusan Losic

School of Chemical Engineering, The University of Adelaide, Adelaide, SA 5005, Australia

E-Mail: dusan.losic@adelaide.edu.au

Editorial Board Member

Prof. Dr. Vladimir M. Mirsky

Faculty of Natural Sciences / Nanobiotechnology, Brandenburg University of Technology Cottbus-Senftenberg, 01968 Senftenberg, Germany

E-Mail: Vladimir.Mirsky@HS-Lausitz.de

Editorial Board Member

Dr. Antonella Sola

Department of Engineering “Enzo Ferrari”, University of Modena and Reggio Emilia, 41125 Modena, Italy

E-Mail: antonella.sola@unimore.it

